# Spectroscopic analysis of embryo culture media for predicting reproductive potential in patients undergoing in vitro fertilization

**DOI:** 10.4274/tjod.92604

**Published:** 2017-09-30

**Authors:** Ercan Baştu, Uğur Parlatan, Günay Başar, Harika Yumru, Nima Bavili, Fatih Sağ, Sibel Bulgurcuoğlu, Faruk Buyru

**Affiliations:** 1 İstanbul University İstanbul Faculty of Medicine, Department of Obstetrics and Gynecology, İstanbul, Turkey; 2 İstanbul Technical University Faculty of Science and Letter, Department of Physics Engineering, İstanbul, Turkey

**Keywords:** In vitro fertilization, embryo culture media, morphology, Raman spectroscopy

## Abstract

**Objective:**

To predict the reproductive potential of embryos via Raman spectroscopy evaluation of the spent culture media as well as with a conventional morphologic evaluation.

**Materials and Methods:**

Women of reproductive age (n=31) who were treated for unexplained infertility and scheduled for single embryo transfer were invited to participate in this prospective study. After the embryos were removed from the culture, the spent culture media were stored at -80 °C after snap-freezing in liquid nitrogen.

**Results:**

Fifteen patients were clinically pregnant, and 16 patients were clinically non-pregnant. Clinical pregnancy was predicted using Raman spectroscopy in 93% (14/15) of clinically pregnant patients, and in 62.5% (10 out of 16) of clinically non-pregnant patients. The sensitivity of the Raman spectroscopic analysis was 93% and the specificity was 62.5%.

**Conclusion:**

Metabolomic evaluation of spent embryo culture media is an emerging technique with promising objective results. However, there is clearly room for improvement.

## PRECIS:

Metabolomic evaluation of spent embryo culture media is an emerging technique with promising objective results; however, there is clearly room for improvement.

## INTRODUCTION

The identification of embryos with the highest potential to implant and establish ongoing pregnancy is the primary aim in human-assisted reproduction. This task is undertaken every day by embryologists worldwide during the treatment of couples that wish to conceive through in vitro fertilization (IVF). The optimal scenario is the transfer of a single embryo that gives rise to a singleton pregnancy. However, even in patients with good prognosis, namely patients aged under 35 years, implantation and the subsequent success rates are well below the expectations of infertile couples undergoing IVF treatment. Therefore, multiple embryo transfer has become clinical routine in IVF clinics worldwide to increase the rate of pregnancies. This approach has consequences such as an increased rate of multiple gestations, which are regarded as high-risk pregnancies^(1)^. Furthermore, the treatment process may cause considerable emotional, financial, and physical distress for couples undergoing IVF treatment because nearly two out of every three IVF cycles do not result in pregnancy^(2)^. The current limitations in the determination of embryos that have the highest implantation potential probably contribute to the low rates of pregnancy during IVF treatment. Hence, improvement of embryo selection has been a “hot research topic” since the beginning of IVF.

Morphology has been a very obvious parameter to assess embryos because it provides a chance for their evaluation from the oocyte stage, all the way to the blastocyst stage. Thus, in the first era of IVF, there was a number of studies that evaluated this parameter and associated morphology with IVF success rates. On the other hand, some researchers stated that the slight increase in pregnancy rates during IVF treatment was most likely a result of better practice in the laboratory than morphologic evaluation^(3)^. Due to the limitations of morphologic evaluation, several researchers investigated adjunctive non-invasive approaches for the assessment of the embryo, such as metabolic parameters^(4)^.

Raman spectroscopy is a vibrational technique that has been used in the field of metabolomics, as well as other techniques such as near-infrared (NIR) spectroscopy, mass spectroscopy, and various others. Raman techniques use a laser to gather vibrational strokes or anti-strokes together with vibrations from the analyzed molecules. Laser-illuminated samples produce scattered light. The amount of light absorbed at a number of vibrational frequencies is then calculated. In the field of metabolomics, one advantage of the Raman technique is that evaluation of liquid samples is straightforward because water gives out weak signals.

The purpose of the present study was to predict the reproductive potential of embryos through Raman spectroscopy evaluation of the spent culture media, as well as conventional morphologic evaluation. We hypothesized that adding Raman spectroscopy to morphologic evaluation would predict better results than those of conventional morphologic evaluation alone.

## MATERIALS AND METHODS

### Patients

Women of reproductive age (18 to 35 years) who were treated for unexplained infertility in the Infertility Clinic of İstanbul University Faculty of Medicine (İstanbul, Turkey) were invited to participate in this prospective study. Inclusion criteria for the diagnosis of unexplained infertility were as follows: (1) duration of infertility for >1 year; (2) confirmation of regular menstrual cycle, (2) hysterosalpingogram-confirmed presence of normal tubal patency; (3) normal day-3 hormonal panel of follicle-stimulating hormone and estradiol, and total antral follicle count more than 7 (4); normal semen analysis results according to the 2010 World Health Organization criteria(3); (5) planned to undergo IVF/intracytoplasmic sperm injection (ICSI) treatment; and (6) planned to undergo day-3 embryo transfer. This study was approved by Ethics Committee of İstanbul University Faculty of Medicine (2015) and informed consent was received from all participants.

All patients received the same ovulation stimulation and monitoring protocols as previously described^(5)^. In brief, the patients underwent ovarian stimulation with a conventional gonadotropin-releasing hormone antagonist protocol.

After oocyte retrieval, cumulus complexes were isolated in the embryology laboratory and mechanically stripped. Afterwards, oocytes were put into separate 50 mL of culture (Sagew, Quinn’s advantage protein plus cleavage medium, Cooper Surgical, Inc., Trumbull, CT, USA). The same culture was used in all embryos. Conventional ICSI was used in all patients according to our laboratory’s process. After the presence of fertilization was identified on day-2 and day-3, as assessed by morphologic evaluation, developed embryos were put into separate 50 mL of culture until the cleavage stage. Throughout the study, standard tri-gas incubators that provided a 5% oxygen environment were used and all embryos were cultured in separate media.

### Morphologic evaluation

Grade-1 embryos were transferred and analyzed according to the morphologic classification as established by Depa-Martynow et al.^(6)^. Grade-1 was considered as embryos with ≥7 blastomeres similar in shape and with <20% of cytoplasmic fragmentation.

### End-point

Clinical pregnancy was the primary outcome of the study, which was defined as the presence of a fetal heartbeat using vaginal ultrasound at 6 weeks of amenorrhea.

### Study design

After the embryos were removed from the culture and prepared for transfer, the spent culture media were loaded into separate labeled cryovials (Nunc Intermed, Kamstrup, Denmark), and stored at -80 °C after snap-freezing in liquid nitrogen. Cryovials were shipped on dry ice to the Raman Spectroscopy Laboratory at İstanbul Technical University (İstanbul, Turkey).

### Raman spectroscopy

The thirty-one samples of 31 patients that were shipped to the Raman Spectroscopy Laboratory for measurement were kept at room temperature for a few minutes and then poured into custom-designed disk-shaped sample cells. The liquid samples were filled in a volumetric cylinder with a diameter of 1.6 mm and length of 6 mm. These cells allowed small volumes with a reservoir of 30 μL and consequently resulted in a long optical path for Raman scattering. The samples were exposed to a single longitudinal mode, 785 nm diode laser, whose output power was 100 mW. Inelastically-scattered photons were collected with a lens (scattering in 1800 geometry) and then focused into the 100-µm entrance slit of a spectrograph with another lens. The inserted photons were dispersed with a grating (600 l/mm) according to the wavelengths in the spectrograph and were imaged on a charge-coupled device (CCD) camera.

The spectra to be formed on the CCD camera, which were the multiple images of the entrance slit, were registered. Measurements were performed sequentially over 10 minutes. After a cleaning procedure, a distilled water spectrum was measured as background before every measurement in the same sample cell. A toluene spectrum was measured after every spent culture measurement in order to check laser stability and to have a reference for Raman calibration.

### Spectral and statistical analyses

**Figure 1:**
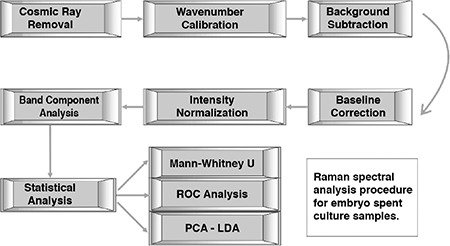
Raman spectral analysis procedure for embryo culture samples ROC: Receiver operating characteristic, PCA: Principal component analysis, LDA: Linear discriminant analysis

The Raman spectra were preprocessed using homemade software written on MATLAB and Simulink software (Mathworks, Natick, MA, USA). The spectral analysis preprocess steps are summarized in Figure 1. In brief, the spectra were first cleaned of unwanted cosmic ray peaks. After calibration of the wavenumbers, water spectra were subtracted from the embryo culture measurements as background correction. The residual fluorescence background profile of each spectrum was corrected using a third order polynomial and normalized to their maximum intensity.

**Figure 2:**
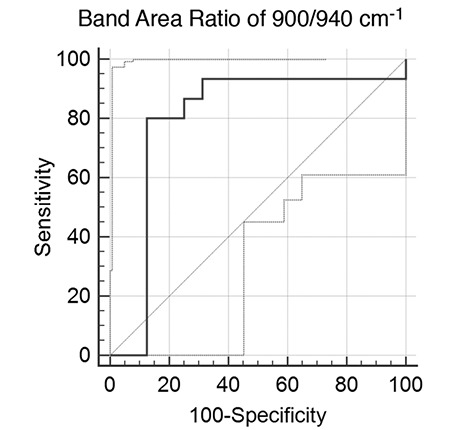
Receiver operating characteristic analysis was made for the band area of 900/940 cm^-1^. The faint lines show the 95% confidence interval

Band component analysis was applied on the preprocessed spectra for 815-1065 cm^-1^ and 1140-1500 cm^-1^ regions using Gaussian line profiles. The bands were statistically analyzed in view of the pregnancy rates. Each band was tested using the Mann-Whitney U test. Only the band ratio of 900/940 cm-1 was statistically significant (p<0.5) (Figure 2).

Principal component analysis (PCA) together with quadratic discriminant analysis (QDA) were applied to the measurements for regions containing 900 and 940 cm-1 bands. The numbers of variables were picked as low as possible because PCA is affected by low sample numbers. The analysis was performed for a partial region of the spectra between 890 and 950 cm^-1^ where the most significant bands found in the band component analysis were located. The first two principal components were used as inputs for QDA analysis. A leave-one-out cross validation was applied to the QDA classifiers in order to obtain the best model. Receiver operating characteristic (ROC) analysis was performed on the QDA classifiers to test the accuracy of the analysis and to find a cut-off value between the two groups.

A sample size calculation was not conducted a priori because previous studies reported no significant differences between groups that were analyzed with conventional morphology and spectroscopic methods^(7)^.

## RESULTS

Thirty-one spent embryo culture media from 31 patients who met the inclusion criteria of the study were preprocessed.

**Figure 3:**
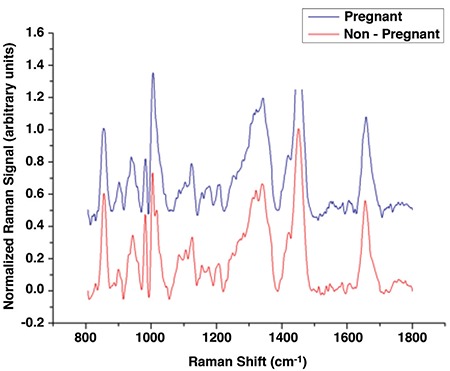
Raman spectra of two selected samples are demonstrated. The corresponding spectra of embryos that developed to pregnancy were manually shifted for better visualization

The averages of Raman spectra were calculated for the pregnant and non-pregnant groups. The average spectra are demonstrated in Figure 3 for group comparison. The spectra show no clear distinction but was shifted for better visualization between the samples of patients whose embryos developed to pregnancy or not.

**Figure 4:**
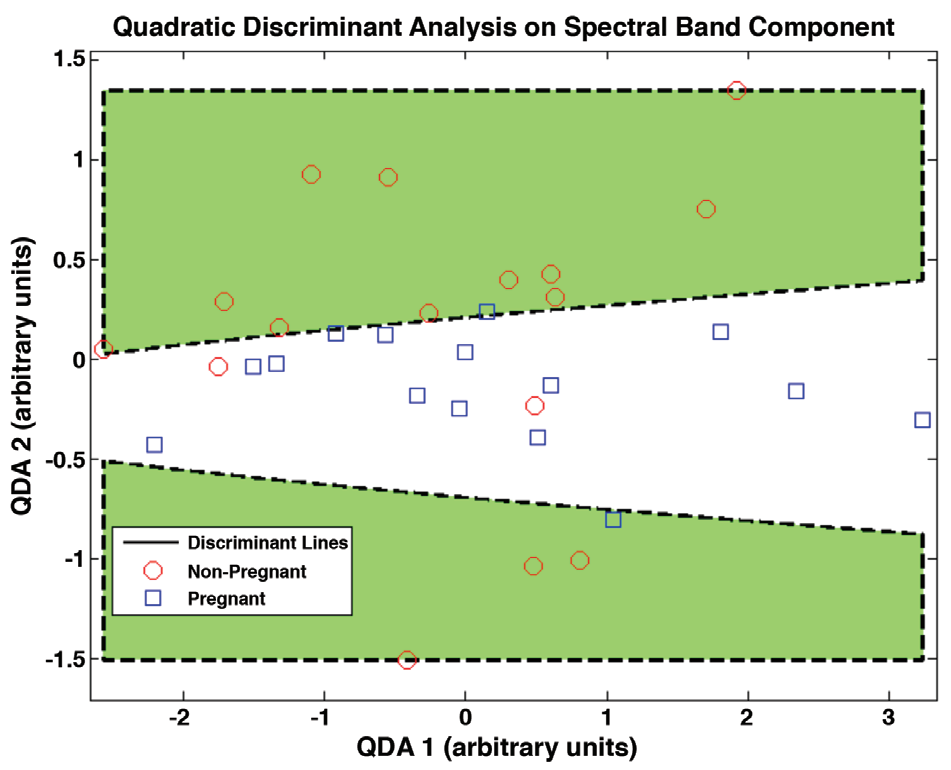
Quadratic discriminant analysis on the band area ratios obtained from band component analysis of the Raman spectra of 31 samples. Discriminant lines were calculated using home-made software that minimizes within-group distances and maximizes between-group distances QDA: Quadratic discriminant analysis

The similarity between the groups described above underscores the need for sophisticated statistical methods such as Raman spectral analysis. We applied QDA after PCA for the region between 890 and 950 cm^-1^. The score plot of this analysis is shown in Figure 4. Each discriminant line is a parabola that maximizes the between-group distance and minimizes the within-group distance because QDA is a discriminant analysis method that uses second order polynomial function.

**Figure 5:**
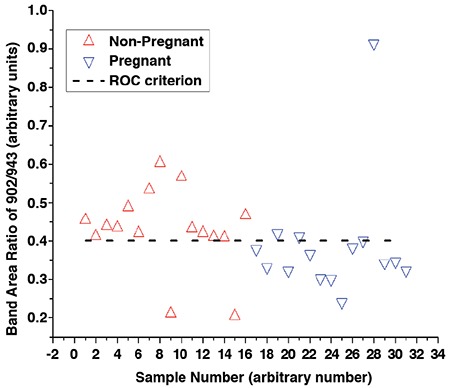
Receiver operating characteristic analysis was performed and a threshold of 0.4007 was found ROC: Receiver operating characteristic

The accuracy of PCA-QDA analysis was tested with an additional ROC analysis. The curve obtained from the analysis is shown in Figure 5. ROC analysis was performed and a threshold of 0.4007 (band area ratio) was found. This curve shows that the optimal specificity and sensitivity of this accuracy ROC analysis was 80.25% and 87.50%, respectively.

Fifteen patients were clinically pregnant, and 16 patients were clinically non-pregnant (Table 1). Clinical pregnancy was predicted using Raman spectroscopy in 93% (14/15) of clinically pregnant patients, and in 62.5% (10 out of 16) of clinically non-pregnant patients. The sensitivity of the Raman spectroscopic analysis was 93% and the specificity was 62.5%.

## DISCUSSION

The present study identified that adding Raman spectroscopic analysis of spent embryo culture media revealed that this approach may predict clinical pregnancy as an adjunct to morphologic evaluation.

Currently, conventional morphologic evaluation is the most commonly used approach in assessing embryo quality worldwide. It has been used since the beginning of IVF, first as a tool to explain embryo development^(8)^, and then to select the embryo with the highest implantation transfer potential^(9,10,11,12,13)^. Morphologic evaluation is based on the embryologist; therefore, embryo scores can vary considerably because it is still a somewhat subjective matter^(14)^.

Metabolomics is an emerging “omics” science that has evolved from proteomics, genomics and transcriptomics. It systematically analyzes the inventory of metabolites. These metabolites are representatives of functional phenotypes at a cellular level. Within this context, researchers focused on the composition of the culture media. The subsequent step was the metabolomic analysis of what embryos use from the culture media and release during their development and to understand if there was a correlation with successful or unsuccessful implantation to the maternal uterus^(15)^.

Seli et al.^(16)^ documented the first proof-of-concept study, which correlated the metabolome of the spent embryo culture media with embryo viability using Raman spectroscopy. The mean spectrum of embryos that did not implant was compared with the mean spectrum of embryos that implanted successfully. In a study by Scott et al.^(17)^, the mean spectrum was validated by analyzing spent embryo culture from a different IVF center that used a different type of culture. Further studies with either Raman and/or NIR spectroscopy argued that the metabolomic profile of the spent embryo culture media was a parameter that was independent of embryo morphology^(18,19,20,21,22,23,24)^. The present study is in line with these findings and shows that metabolomic evaluation of spent embryo culture media alone can predict reproductive potential as efficiently as conventional morphologic evaluation of the embryo.

Interestingly, despite the promise of metabolomic evaluation, the use of Raman spectroscopy and/or other techniques such as NIR and mass spectroscopy have limited clinical presence. There are a few potential explanations for these limitations. Currently, the equipment used for these spectroscopic techniques are expensive and require dedicated specialists. Moreover, prompt results are needed during embryo transfer in clinical practice and the applicable information gathered from spectroscopic techniques still requires time. If we look specifically at Raman spectroscopy for the purpose of metabolomic evaluation, it provides weak signal intensity, which may be a drawback when sample concentrations are low. Enhancement methods such as surface-enhanced Raman spectroscopy (SERS) or resonance Raman spectroscopy (RRS) can be used to overcome this limitation. On the other hand, RRS may damage the sample and produce broad background in the spectra; therefore, it may shield information from the spectra. Although SERS suffers from repeatability, it is a developing method and may be useful for future studies. However, it is important to note that even with the aforementioned drawbacks, Raman spectroscopy alone still produced results that were comparable to conventional morphologic evaluation in the present study. Hence, if these drawbacks were to be overcome in future studies, metabolomic evaluation could provide a more objective approach to the current morphologic evaluation and possibly result in becoming a more accurate technique with increased reproductive outcomes.

### Study Limitations

A limitation of the study was its small sample size. We wanted to create an ideal design by only including single embryo transfer. Due to the financial aspects of IVF treatment and psychological burden, single embryo transfer is only occasionally feasible. Also, Raman spectroscopy equipment use and evaluation of embryo culture media required time to give results that could be utilized in routine clinic practice.

## CONCLUSION

Existing embryo assessment relies heavily on the morphologic evaluation of the embryo by an embryologist. Furthermore, this subjective method does not provide sufficient specificity or sensitivity to produce desirable pregnant rates for patients receiving IVF treatment. Metabolomic evaluation of spent embryo culture media is an emerging technique with promising objective results. However, there is clearly room for improvement in the exact spectroscopic technique used for metabolomic evaluation. Moreover, it has to be adequately validated. Hence, this approach still remains experimental and its application has not translated into the clinical setting. Further randomized control trials with improved spectroscopic techniques are needed to document the potential benefit from the use of metabolomic evaluation alone or as an adjuvant approach to the conventional morphologic evaluation.

## Figures and Tables

**Table 1:**
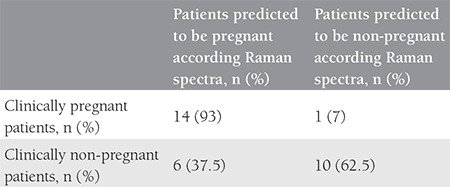
Clinical pregnancy rates according to conventional morphologic evaluation and Raman spectra analysis
